# Fertility preferences and unmet need for family planning in women with multiple sclerosis

**DOI:** 10.3389/fneur.2022.1035596

**Published:** 2022-11-09

**Authors:** Lorena López-Reyes, Claudia Guío-Sánchez, Catalina González-Uribe, Simón Cárdenas-Robledo

**Affiliations:** ^1^Departamento de Neurología, Centro de Esclerosis Múltiple Hospital Universitario Nacional de Colombia, Bogotá, Colombia; ^2^Escuela de Medicina, Universidad de los Andes, Bogotá, Colombia; ^3^Departamento de Neurología, Universidad Nacional de Colombia, Bogotá, Colombia

**Keywords:** multiple sclerosis, fertility preferences, needs assessment, contraception, needs assessment (health services accessibility)

## Abstract

**Background:**

Most women with multiple sclerosis (MS) have childbearing potential. Although fertility and pregnancy are not affected by MS, the fertility preferences of women with MS can change due to the risk of complications for the mother and/or adverse pregnancy outcomes resulting from the disease or its treatment.

**Objectives:**

To describe fertility preferences (FPs) and their associated factors, to estimate the Unmet Need for Family Planning (UNFP), use of contraceptives, and history of exposure to disease-modifying therapies (DMTs) during pregnancy in women with MS.

**Methods:**

In a cross-sectional observational study, a random sample of women with MS were surveyed with the FP subset of the Demographic and Health Survey of Colombia. Factors associated with FP were evaluated through bivariate and logistic regression analysis. The proportion of pregnancies exposed to DMTs, UNFP, and use of contraceptives was estimated.

**Results:**

Of the 141 women interviewed, 101 women had childbearing potential, of whom 49 did not want to have children, 38 were sterilized, 33 wanted to have more children, 19 were undecided, and 2 stated they were unable to bear children (menopause or hysterectomy). No MS-related variables were associated with the preference to have more children. Age (OR 0.91; 95% CI 0.84–0.98) and the number of children (OR 0.23; 95% CI 0.09–0.58) decreased the likelihood of desire for children. Of 116 sexually active women, 87.06 % (101) were using contraceptives, and among them, four were using fertility awareness methods and withdrawal. The UNFP was estimated at 6.03% and was not significantly different from the general population. Eighty-two pregnancies were identified, of which 48 occurred after diagnosis, and 25 were exposed to DMTs.

**Conclusion:**

Fertility preference in women with MS is not associated with clinical variables. A large proportion of women choose not to have children and prefer to use permanent contraceptive methods. Although the frequency of contraceptive use was high, some women have the UNFP and use low-efficacy contraceptive methods, which may result in unplanned pregnancies.

## Introduction

Multiple sclerosis (MS), a chronic, inflammatory, and neurodegenerative disease, is the leading cause of non-traumatic disability in young adults and mostly affects young women with childbearing potential (1).

Women with MS report disease-related concerns in several areas that may influence their pregnancy desire. These can occur in relation to future offspring, such as the risk of inheriting MS (2, 3), the risk of harm to the fetus or newborn/infant by exposure to disease-modifying therapies (DMTs) (3, 4), and limitations in the ability to care for the children due to disability and MS symptoms (2–6). In relation to MS itself, the concerns described include the unpredictability of MS (4, 7, 8). Finally, the lack of social support has also been described as an important issue for women with MS when deciding to get pregnant (6).

According to a systematic review and meta-analysis of pregnancy and fetal outcomes in women with MS, interferons, glatiramer acetate, and natalizumab do not appear to increase the risk of spontaneous abortions, pre-term birth, or major congenital malformations (9, 10). Regarding teriflunomide [category X (FDA)/category 1 (EMA)] and fingolimod [category C (FDA)/category 2 (EMA)], there are absolute contraindications for use during pregnancy from both the US Food and Drug Administration and European Medicines Agency.

Therefore, it is necessary to be aware of the desire of women with MS to become pregnant prior to the starting DMTs, and during the course of the disease, as well as encourage them to plan pregnancy. Defining a safe pregnancy plan will depend on MS activity and the DMTs being used (9–11). On the contrary, if there is no pregnancy plan and women are using DMTs, the use of contraceptive methods is advised (12, 13). No negative impact on the course of MS associated with the use of contraceptives has been reported (14, 15). Long-acting, reversible contraception (LARC) may be particularly effective, as they do not require proactive compliance by women with MS (13, 16).

At a global level, according to studies developed to determine the risk of MS (17) associated with the use of oral contraceptives (OCP), the use of OCPs has been reported in Portugal in 54/132 (40.9%), which turned out to be lower when compared with women in the USA, with 46/162 (28.3%) (14). Finally, in the Spanish population, 78.6% reported having used OCPs (15). In Colombian women aged 13–49 years, according to the 2015 Demographic and Health Survey of Colombia (DHS), 81.7% were using contraceptive methods (18). For Colombian women with MS in 2019, we reported that only 58.5% were using contraceptives (19).

The Unmet Need for Family Planning (UNFP) is defined as the proportion of women who have childbearing potential and are sexually active and who report not wanting any more children or wanting to delay the next child, but are not using any method of contraception (20). The UNFP is considered a measure of the success of reproductive health programs and is one of the indicators of the Millennium Development Goals. The concept of UNFP points to the gap between women's reproductive intentions and their contraceptive behavior (21).

In the latest DHS in 2015 in Colombia (18), the UNFP for women in the general population was estimated at 5.5%, while the total UNFP for women living in the capital city (Bogotá) was estimated at 3.2%. The UNFP in sexually active unmarried women was 8.0% and in married women 3.7% (18). The UNFP has been barely studied in populations with special needs, such as those with chronic diseases (22, 23) including MS (24).

Therefore, in view of the concerns about pregnancy, the low frequency of contraceptive use in our population, and limited data of the UNFP in women with MS, we aimed to describe the fertility preferences and their associated factors in order to estimate the UNFP and describe the history of exposure to DMTs during pregnancy in women with MS cared for at our Center.

## Materials and methods

This is a cross-sectional observational study. Eligible women were 18–45 years old and had MS confirmed with current criteria (25), who attended consultations at our MS Center. Data collection was conducted during the COVID-19 pandemic period from June 2020 to March 2021. Subjects with severe dysarthria or cognitive impairment that could preclude answering the surveys (as reported by the clinical charts) were excluded.

Women were invited to participate, and those who accepted were interviewed by one of the authors (LL-R) who performed the surveys. This had to be done *via* telephone, due to the restrictions imposed during the COVID-19 pandemic. Demographics and basic clinical characteristics and history of exposure to DMTs during pregnancy were collected during the interviews and from our cohort's database. We used the items from the fertility preferences section from the 2015 DHS (16). These items include the desire to have children and their timing, as well as the use of contraceptives. This survey is representative on a national level in Colombia.

### Variables

#### MS clinical variables

The clinical variables obtained from the clinical records were the clinical phenotype, categorized according to the current classification (26) into relapsing MS [RMS—including those patients who had had only one clinical episode (CIS)] and progressive MS (PMS—both primary and secondary progressive MS); time since diagnosis, according to date of diagnosis and the moment of the data analysis and calculated in years; the total number of relapses since the onset of the disease and the number of relapses in the year prior to the study; and disability, assessed with the Expanded Disability Status Scale (EDSS) (27). This scale attempts to quantify the neurological deficit observed in the different functional systems affected in MS and is scored from 0 (normal neurological examination) to 10 (death from MS). Treatment was described according to the DMTs used at the time of the surveys.

#### Demographic variables

Age, education attainment, marital status, and socioeconomic level were analyzed. Education attainment was classified as basic (primary and secondary education) and high level (post-secondary education). The socioeconomic level is assigned according to the place of residence in six strata (with stratum 1 being the lowest and stratum 6 being the highest) and was categorized into three groups: low (strata 1 and 2), middle (strata 3 and 4), and high (strata 5 and 6) (28). Marital status was categorized into women who are married or in a common law marriage, and unmarried women, including single, widowed, and divorced women.

#### Fertility preferences

The question on fertility preferences from the DHS is framed as, “Would you like to have a/another child with your husband/partner, or would you prefer not to have any more children?” Responses to this question were categorized into: “want a (another) child,” “want no more,” “cannot get pregnant,” “undecided,” and “don't know” (18). Our outcome variable was categorized into i) women with the desire to have children and ii) women who were sterilized, undecided, and did not want more children. For this analysis, we excluded those who reported that they were unable to have children. The number of children corresponded to the number of living children.

We also surveyed the MS-related reasons that contributed to the women's decision to not have any children or those who were undecided.

#### Unmet need for family planning

We calculated the prevalence of the UNFP using the adapted algorithm proposed by Bradley et al. (20). To estimate the UNFP in women with MS, we modified the algorithm to group married and single women, because our interest was in assessing the risk of pregnancies in sexually active women, regardless of marital status. This algorithm is also used to estimate the frequency of contraceptive use which is classified into two groups (20): limiting methods (sterilization) and pregnancy spacing methods (intrauterine device, subcutaneous implant, pill, injectables, vaginal ring, male condom, fertility awareness-based methods [FAMs], and withdrawal) (29). This latter group of women were analyzed as women who were not using contraceptive methods (30).

Women who do not use contraceptives were identified based on the question, “Are you or your partner currently doing anything or using any method to delay or avoid pregnancy?”

From this group, for the UNFP calculation, the following were excluded:

- Those who, in response to the question, “Would you like to have another child or would you prefer not to have any/more children?”, answered that they wanted to have children in <2 years.
- Those who, in response to the question, “Are you currently pregnant?”, answered yes.
- Women who, in the reasons for not using contraceptives, responded: i. they are not sexually active, ii. they have sex with women, iii. menopause, and iv. hysterectomy.

Pregnant women who answered affirmatively to the question “When you became pregnant, did you want to become pregnant at that time, did you want to have a child later, or did you not want children?” were considered to be willing to get pregnant.

#### History of exposure to DMTs during pregnancy

We described the number of pregnancies after the diagnosis, after treatment onset, and the time in months of exposure to DMTs during pregnancy. We also asked whether the patients discussed with a neurologist the risks associated with DMTs and pregnancy before the pregnancy took place.

### Data analysis

Associations between the desire to have children and categorical and continuous variables were assessed using the Fisher's exact and Wilcoxon rank sum tests, respectively. The variables with *p*-values lower than 0.05 were considered for the multivariable logistic regression.

The model with the best fit based on the Akaike information criterion (AIC) was chosen. To assess the presence of an association between the desire to have children and independent variables, a *p*-value of <0.05 in the final model was considered statistically significant. Finally, the goodness of fit of the model was ascertained from the ROC curve, sensitivity, and specificity of the model.

The decision not to have any children or being undecided in relation to MS, UNFP, use of contraceptives, and history of exposure to DMTs during pregnancy were described in terms of absolute and relative frequencies. To determine the difference between the proportion of the UNFP between women with MS and women from the general population in our city, we used the two-sample proportion test. The confidence intervals were estimated using the Wilson score interval test.

The women invited to participate were chosen randomly. The sample size was estimated under the assumptions of a proportion of 50%, a precision error of 5%, a confidence interval (CI) of 95%, and based on the number of active patients in our cohort until March 2020. Assuming a non-respondent rate of 15%, a sample size of 141 women was calculated.

The study data were gathered and managed using REDCap, and we used STATA statistical software for the analysis (version 15, StataCorp LLC, College Station, TX, USA).

## Results

**Figure 1:**
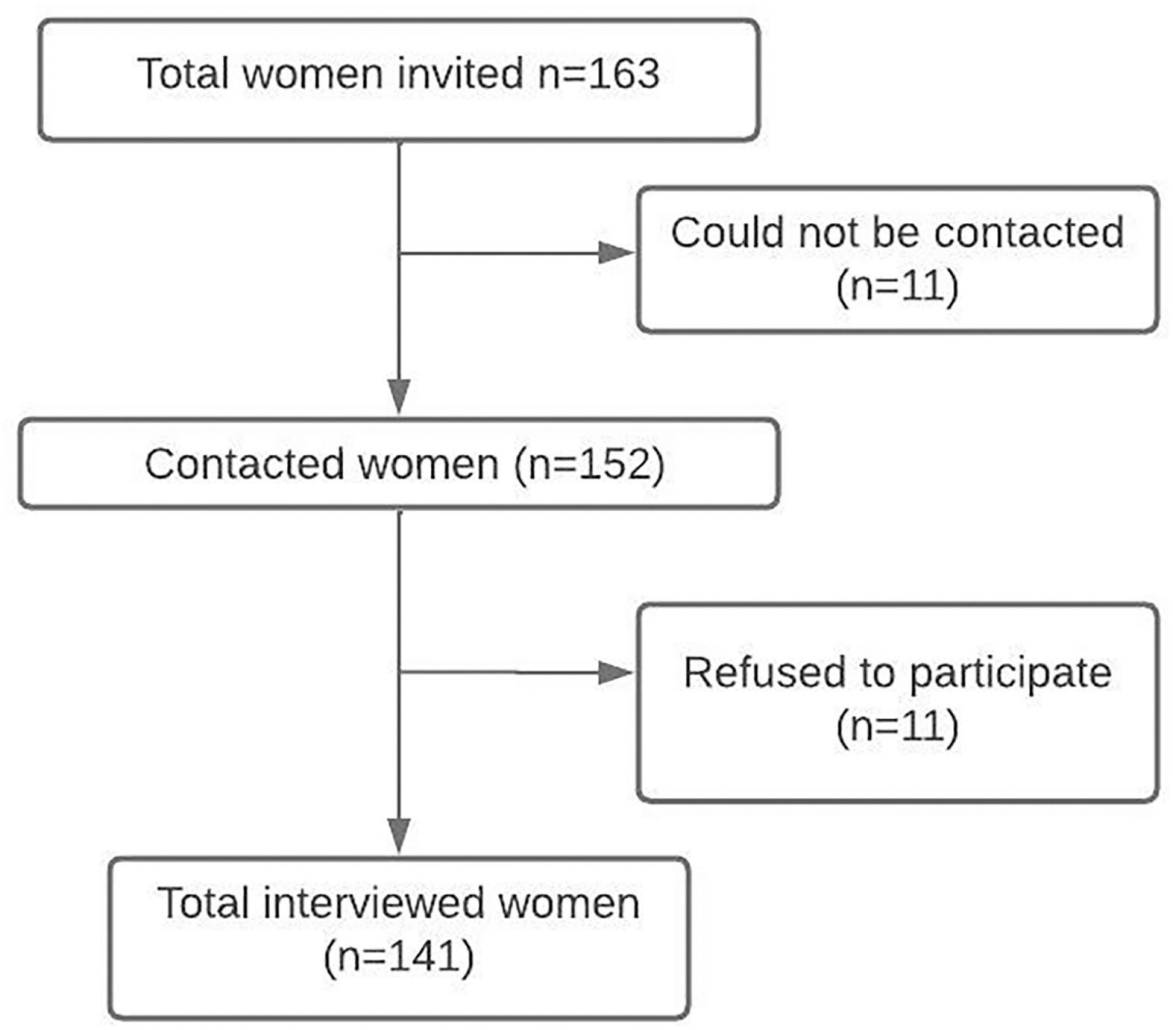
Study sample flowchart of women with MS surveyed.

A total of 163 women were invited to participate in the study, of whom 11 could not be contacted and 11 refused to participate. No missing data were found for analysis (Figure 1).

### Demographic and clinical variables

Table 1 shows the demographic and clinical characteristics of the 141 women studied. The median (IQR) age was 37 (10) years. One hundred and twenty-eight (90.78%) had a high level of education. Regarding socioeconomic status, 22 (15.6%) were in the low category, 98 (69.5%) were in the middle category, and 21 (14.89%) had a high socioeconomic status. Eighty-two (58.16%) of the respondents were married or had a common law marriage.

**Table 1:** Demographic and clinical variables.

**Variable**	**(*n* = 141)**
**Age, median (IQR)**	37 (10)
**Education attainment**, ***n (%)***	
Secondary	13 (9.22)
Higher	128 (90.78)
**Socioeconomic level**, ***n (%)***	
Low	22 (15.6)
Middle	98 (69.5)
High	21 (14.89)
**Marital status**, ***n (%)***	
Married/in-union	82 (58.16)
Never married/widowed/divorced/separated	59 (41.84)
**Clinical phenotype**, ***n (%)***	
Relapsing–remitting	134 (95.04)
Progressive	4 (2.84)
Clinically isolated syndrome (CIS)	3 (2.13)
**Age at diagnosis years, median (IQR)**	29 (10)
**Time at diagnosis years, median (IQR)**	6 (8)
**Disease-Modifying Therapies (DMT)**, ***n*** **(%)**	
Fingolimod	44 (31.21)
Natalizumab	22 (15.60)
Alemtuzumab	14 (9.93)
Interferons	13 (9.22)
Dimethyl fumarate	13 (9.22)
Anti-CD20 therapies	12 (8.51)
Glatiramer acetate	8 (5.67)
Without DMT	8 (5.67)
Teriflunomide	7 (4.96)
**EDSS**, ***n*** **(%)**	
0.0–2.5	122 (86.52)
3.0–5.5	16 (11.35)
> 6.0	3 (2.12)
**Relapses during the last year**, ***n*** **(%)**	20 (14.18)
**Total number relapses, n (%)**	
One relapse Two relapses Three relapses Four relapses Five relapses Six relapses	49 (34.75) 33 (23.40) 23 (16.31) 11 (7.80) 10 (7.09) 15 (10.64)

Most women (95.04%) had RMS. The median (IQR) age at diagnosis was 29 (10) years, and the median (IQR) time since diagnosis was 6 (8) years. One hundred and thirty-three patients (94.3%) were treated with DMTs. One hundred and twenty-two women (86.52%) had an EDSS score between 0.0 and 2.5, 16 (11.35%) between 3.0 and 5.5, and 3 (2.12%) higher than 6.0. Twenty women (14.18%) reported relapses in the previous year.

### Fertility preferences

The desire not to have children was reported by 49 (47.57%) of the women interviewed. Thirty-eight (26.95%) were sterilized, 33 (23.40%) wanted to have more children, 19 (13.48%) were undecided about having children, and 2 (1.94%) stated they were infertile. Seventy (49.65%) of the participants did not have children, 43 (30.50%) had one child, 23 (16.31%) reported having two children, and 5 (3.55%) had three or more children.

Age (*p* < 0.001), level of education (*p* = 0.046), age at diagnosis (*p* < 0.001), and the number of children (*p* < 0.001) were selected for the logistic regression models (Table 2). Based on the lowest AIC, the final model included age, number of children, and level of education (Supplementary material).

**Table 2:** Bivariate analyses (*N* = 139).

**Variable**	***p*-value**
Age^a^	<0.001
Education attainment^b^	0.046
Socioeconomic level^b^	0.910
Marital status^b^	0.317
Clinical phenotype^b^	0.209
Age at diagnosis^a^	0.003
Time diagnosis^a^	0.551
EDSS^b^	0.825
Relapses at last year^b^	0.092
Total number relapses^b^	0.569
Number of children^a^	<0.001

a Fisher's exact test;

b Wilcoxon rank sum test.

The logistic regression analysis showed that age (OR 0.91; 95% CI 0.84–0.98; *p* = 0.021) and number of children (OR 0.23; 95% CI 0.09–0.58; *p* = 0.002) were associated with a reduced likelihood of wanting to have more children in future (Table 3). The correct classification rate of the model corresponds to 79.14% with a sensitivity of 30.3% and a specificity of 94.34%. The area under the ROC curve corresponds to 83.26%.

**Table 3:** Factors associated with fertility preferences.

**Variable**	**OR**	**SD**	***P* value**	**95%IC**
Age	0.913	0.035	0.021	0.845–0.986
Number of children	0.237	0.109	0.002	0.095–0.585
Basic education	0.205	0.183	0.077	0.035–1.180

#### MS-related reasons for respondents who did not want or were hesitant to have children

Among 68 women who answered that they did not want to have children or were hesitant about it, 35 (51%) indicated that this decision was related to MS. The main reasons expressed were the fear of MS worsening (27.4%), interruption of DMTs (16.4%), the misconception that “children of people with MS are not healthy” (14.28%), the influence of symptoms in the child's upbringing (13.18%), becoming a burden for the partner or children (13.18%), advice from the neurologist (10.98%), and concerns about child care (4.16%).

### Unmet need for family planning

**Figure 2:**
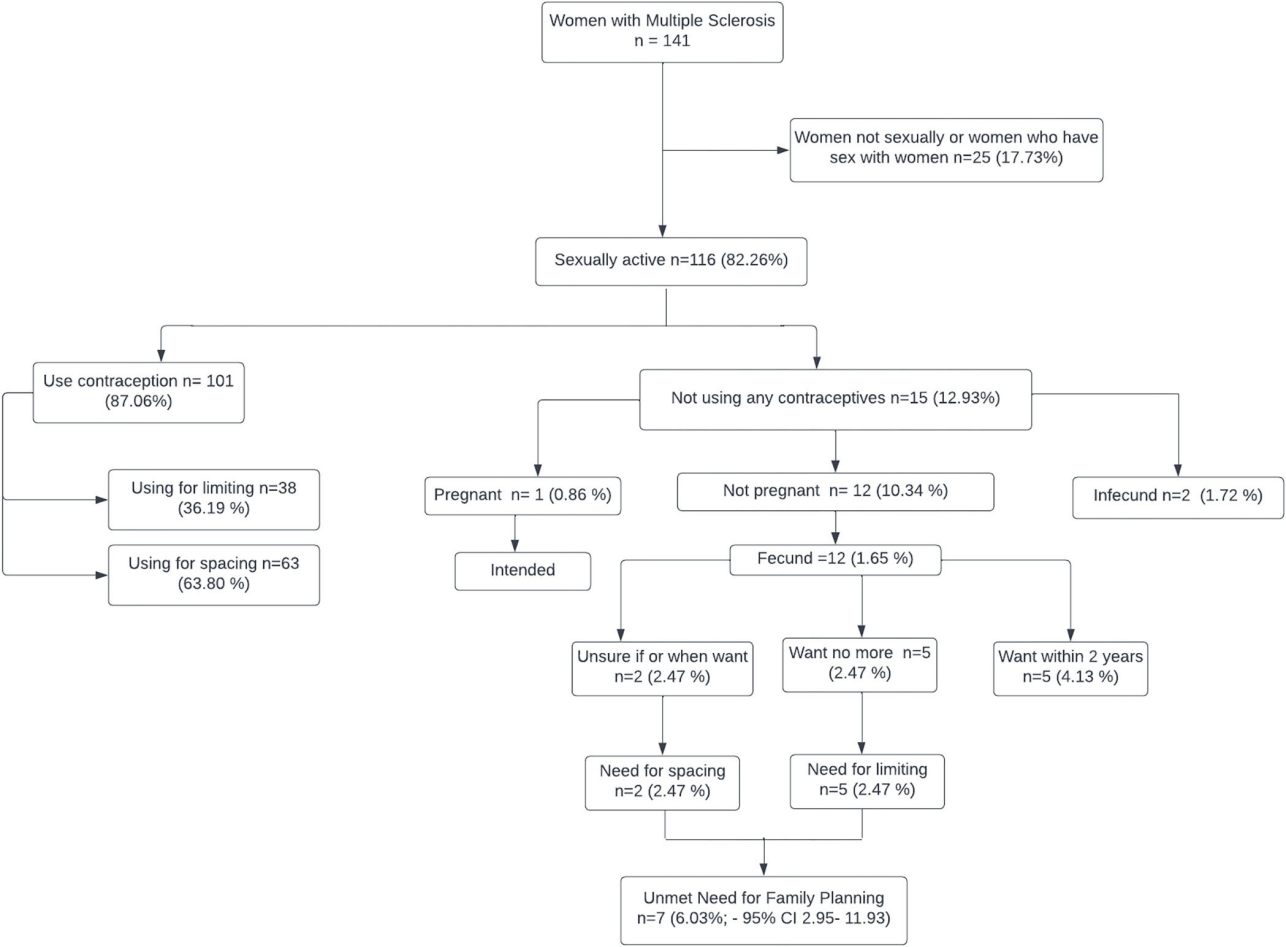
Unmet need for family planning in women with MS.

One hundred and one (87.06 %) women reported using contraceptive methods. Thirty-eight of them (36.19%) used methods to limit further pregnancies, which corresponds to sterilized women. Whether this was the women's choice or a result of medical advice is unknown. Sixty-three (63.80%) used methods to space out having children, of which the most common was the use of condoms, corresponding to 23 women (21.90%) (Table 4). 3.96% of the participants reported the use of FAMs and withdrawal. The UNFP (Figure 2) was estimated at 6.03 % (95% CI 2.95–11.93). The prevalence of the UNFP was not statistically different from that of the general population in our city (*p* = 0.19).

**Table 4:** Contraceptives use (*n* = 101).

**Contraception**	**Family planning methods**	***N* (%)**
Using for limiting	Sterilized	38 (37.62)
Using for spacing	Intrauterine device (IUD)	9 (8.91%)
	Implant	8 (7.92)
	Pill	17 (16.83)
	Injectables	5 (4.95)
	Ring	1 (0.99)
	Male condom	23 (22.77)

### History of exposure to DMTs during pregnancy

Of the 141 women surveyed, 25 (52%) had had exposure to DMTs during pregnancy. Only 8% reported having discussed pregnancy with their neurologist prior to exposure to DMTs.

Twenty-three pregnancies were exposed to DMTs without neurologist medical advice, the majority of these, 19 (82.6%), were exposed to DMTs considered safe in pregnancy such as interferons, glatiramer acetate, and natalizumab. The remaining four (17.39%) cases were exposed to DMTs with a high teratogenic risk and therefore contraindicated during pregnancy. The average exposure time to DMTs during pregnancy was 1 month. Finally, among the four high-risk exposure pregnancies, three had an induced abortion as an outcome and one was an ectopic pregnancy.

## Discussion

This study is the first approach to the outcomes of reproductive health and the identification of unmet needs related to family planning advice for women with MS in our country.

The multivariate analysis showed that no MS-related variables were associated with the preference of having children, but demographic factors such as age and the number of previous children were. This can be explained from the immediate determinants of fertility initially proposed by Bongaarts (31), which were later adjusted by Stover (32). These are defined as biological and behavioral factors that, when interacting with socioeconomic and cultural factors, impact fertility. According to Stover, in this case increasing age as a biological factor decreased the possibility of wanting to have children.

From the standpoint of population studies, a decrease in birth rates when women reach 35 years of age has been systematically observed (33). Likewise, it has been shown that age has an impact on successful conception since as age increases, the possibility of conceiving children decreases proportionally, which is associated with the aging of the ovaries and ovules (34). Therefore, age is a factor that influences reproductive intentions both in the general population and in the population with MS (35).

Our finding of a decreased likelihood of wanting more children for each previous child deserves attention. The reproductive intention in the Colombian population has been shown to be related to cultural and socioeconomic factors such as the number of children, family size, and expectations regarding the ideal number of children (36). We can infer that the number of children is related to the satisfaction of motherhood and thus limits the desire for further reproduction.

We found a high frequency of women who did not want to have children and had permanent contraception, which is in line with previous findings of a lower number of children in women with MS relative to the general population (3, 8). Our findings also suggest that clinical variables such as MS phenotype and number of relapses are not significantly related to the decision to become a mother or not, as previously reported in the Portuguese population (8).

No statistically significant association was found for clinical variables and childbearing preferences to assess the possible concerns of the 51% of women who prefer not to have children or are undecided about having children for MS-related reasons. Our findings are similar to what is known from women with chronic illnesses, who state that the decision to become a mother is difficult and even linked to possible negative health effects (6), which include disease progression, symptoms that could potentially interfere with parenting, and interruption of treatments (2, 5, 8). This would suggest the need to evaluate not only clinical variables, such as those evaluated in this study, but also these women's perceptions of pregnancy.

We found that some women continue to perceive the children of MS patients as unhealthy. This finding was not expected and may be due to healthcare professionals assuming that women have enough knowledge regarding MS, pregnancy, and fetal risks, and not discussing this issue thoroughly. Moreover, these are topics that are discussed with the neurologist prior to starting DMTs. The survey results are therefore consistent with other previous studies. A 2012 study (8) found that among 5,949 participants, the third most common reason for not becoming pregnant was the belief that children would inherit MS (34.7%). A similar study from 2014 found that 21% of MS patients decided to reduce pregnancies after being diagnosed due to the risk of babies inheriting MS (8). Two studies reported that the pregnancy-related concern was related to the child's health (4, 5).

The estimated UNFP for our sample was 6.03%, which is similar to that for the population of women from Bogotá (18). This is relevant for women with an indication for treatment and who are sexually active, which may lead to unplanned pregnancies and, thus, involuntary exposure to DMTs during pregnancy. Another finding to highlight is the use of FAMs, withdrawal, and the high proportion of barrier methods, which, due to their low efficacy and their susceptibility to incorrect and non-systematic use, may lead to the same risk of undesired pregnancies (37).

The proportion of pregnancies that were exposed to DMTs and the outcome of voluntary pregnancy termination for the majority of those exposed to treatments contraindicated in pregnancy are worrisome and may be a reflection of the overall high frequency (52%) of unplanned pregnancies in the Colombian population (38). This has been linked to the lack of use of contraceptive methods, which can occur due to side effects, high costs, infrequent sexual intercourse, lack of availability, and disapproval by the partner, thus leading to their discontinuation, inconsistent or incorrect use, and failure (39).

On the contrary, it has been reported that women who received counseling before becoming pregnant had a lower frequency of unplanned pregnancies (40). This counseling should start with the neurology consultation, as neurologists are recognized by MS patients as the main source of information for pregnancy planning (41).

Therefore, we consider that one of the reasons that may explain exposure to DMTs during pregnancy is the barriers to access specialized neurological care and, with this, inconsistent follow-up. Such barriers may occur due to some features of the Colombian healthcare system, resulting in fragmented care that is linked to the continuity of contracts between insurers and healthcare providers (42).

However, it is possible that the pregnancies reported occurred at a time when access to contraceptive methods and information on pregnancy in MS patients and DMTs exposure was limited, and therefore, the medical practice at that time cannot be judged by current standards.

## Study limitations

Our study has several limitations that must be taken into account. Due to the COVID-19 pandemic, data collection had to be changed from in person to telephone surveys, which creates an information bias. However, the surveys were conducted by a professional with whom the participants had already interacted, so it is possible that this allowed respondents to discuss these sensitive issues more freely, and thus reduced the possibility of apprehension bias.

Another limitation is the selection bias, due to the fact that our specialized center attracts patients with potentially more severe diseases. This is evidenced with the sociodemographic aspects of the surveyed population: 90.78% had high levels of education and only 15.6% had a low socioeconomic status. This differs from the population surveyed nationally in 2015, in which 33.5% of women had high levels of education and 22.6% had a low socioeconomic status (18). Despite our sample being generated randomly, it might not be representative of the MS population in our city. Also, our study might not have been able to detect the effect of some of the variables on the fertility preferences, such as a history of spontaneous or induced abortions and the use of fertility treatments. These are variables that can influence the number of children of the women surveyed and were not taken into account for the fertility preference analysis. However, it should be kept in mind that Colombia is considered to have a low prevalence of MS (43, 44) and that this study sought to approximate the reproductive aspects of MS patients in Bogotá.

## Conclusion

Fertility preferences are not significantly associated with clinical variables, but with the known demographic factors for women in the general population. The majority of women with MS prefer not to have children, and a large proportion of them choose to use contraception to limit pregnancies. The UNFP in women with MS does not differ from that found in the general population, and there is a high frequency of exposure to DMTs during pregnancy. Further studies are needed to address the attitudes of women with MS toward reproductive issues.

## Data availability statement

The raw data supporting the conclusions of this article will be made available by the authors in accordance with the requirements of Research Ethics Committee of Hospital Universitario Nacional de Colombia.

## Ethics statement

The studies involving human participants were reviewed and the Research Ethics Committee of Hospital Universitario Nacional and the Universidad de los Andes approved the study (Ref. 2019-12 and 202001283 respectively). The patients/participants provided their written informed consent to participate in this study.

## Author contributions

LL-R was responsible for study design, study management, and reporting. CG-S, SC-R, and LL-R contributed to the writing of the manuscript. CG-U was academic supervisor, advised on study design, statistical analysis, interpretation, and discussion of results. SC-R and LL-R contributed to the analysis and interpretation of the results. All authors contributed to the article and approved the submitted version.

## Funding

The authors disclose the receipt of financial support for this publication by Roche. This grant was awarded after the research process, data analysis, and the writing of this article.

## Conflict of interest

Author LL-R has received travel expenses for scientific meetings from Roche, Tecnofarma, and Biogen-Idec and speaking honoraria from Merck, Roche, and Biogen-Idec. Author CG-S reports consulting fees from Novartis, Biogen-Idec, Sanofi-Genzyme, Merck, and Roche and has received travel expenses for scientific meetings from Sanofi-Genzyme, Abbot, and Merck. Author SC-R was an ECTRIMS clinical fellowship awardee 2019–2020 and has received travel expenses for scientific meetings from Genzyme and Merck; compensation for consulting services or participation on advisory boards from Merck, Roche, Biogen-Idec, and Novartis; speaking honoraria from Novartis and Biogen-Idec; and research support from Biogen-Idec and Novartis. The remaining author declares that the research was conducted in the absence of any commercial or financial relationships that could be construed as a potential conflict of interest.

## Publisher's note

All claims expressed in this article are solely those of the authors and do not necessarily represent those of their affiliated organizations, or those of the publisher, the editors and the reviewers. Any product that may be evaluated in this article, or claim that may be made by its manufacturer, is not guaranteed or endorsed by the publisher.
