# Supplementary material for: Fertility preferences and unmet need for family planning in women with multiple sclerosis
Source: Front Neurol. 2022 Nov 9;13:1035596. doi: 10.3389/fneur.2022.1035596 (PMC9682175; doi:10.3389/fneur.2022.1035596)
Supplement: Supplementary file 1 [file Table_1.docx]

# Supplementary Material

**Supplementary Table 1 |** **Model selection**

| **Model** | **Variables** | **AIC** |
| --- | --- | --- |
| **M1** | *ln (desire for children) =*  $\beta0+\beta1 (age)+\beta2(number of children) +\beta3 (education attainment)$ | 124.1 |
| **M2** | *ln (desire for children) =*  $\beta0+\beta1 (age)+\beta2(number of children) +\beta3 (education attainment)+\beta4 (age at diagnosis)$ | 125.97 |
| **M3** | *ln (desire for children) =*  $\beta0+\beta1 (age)+\beta2(number of children)$ | 128.65 |
